# Changes in Biomarkers of Exposure on Switching From a Conventional Cigarette to Tobacco Heating Products: A Randomized, Controlled Study in Healthy Japanese Subjects

**DOI:** 10.1093/ntr/nty104

**Published:** 2018-06-15

**Authors:** Nathan Gale, Mike McEwan, Alison C Eldridge, Ian M Fearon, Neil Sherwood, Edward Bowen, Simon McDermott, Emma Holmes, Andrew Hedge, Stuart Hossack, Louise Wakenshaw, James Glew, Oscar M Camacho, Graham Errington, John McAughey, James Murphy, Chuan Liu, Christopher J Proctor

**Affiliations:** 1 British American Tobacco (Investments) Limited, Research and Development, Southampton, UK; 2 Celerion, Inc, Belfast, UK; 3 Neil Sherwood Consulting, Commugny, Switzerland; 4 Early Clinical Services Medical Writing, Global Medical and Regulatory Writing, Covance Clinical Research Unit Limited, Leeds, UK; 5 Early Clinical Development, Covance Clinical and Periapproval Services Limited, Leeds, UK

## Abstract

**Background:**

Smoking is a leading cause of numerous human disorders including pulmonary disease, cardiovascular disease, and cancer. Disease development is primarily caused by exposure to cigarette smoke constituents, many of which are known toxicants. Switching smokers to modified risk tobacco products (MRTPs) has been suggested as a potential means to reduce the risks of tobacco use, by reducing such exposure.

**Methods:**

This randomized, controlled study investigated whether biomarkers of toxicant exposure (BoE) were reduced when smokers switched from smoking combustible cigarettes to using a novel (glo^™^/THP1.0) or in-market comparator (iQOS/THS) tobacco heating product (THP). One hundred eighty Japanese smokers smoked combustible cigarettes during a 2-day baseline period, followed by randomization to either continue smoking cigarettes, switch to using mentholated or non-mentholated variants of glo^™^, switch to using a non-mentholated variant of iQOS, or quit nicotine and tobacco product use completely for 5 days. Baseline and post-randomization 24-h urine samples were collected for BoE analysis. Carbon monoxide was measured daily in exhaled breath (eCO).

**Results:**

On day 5 after switching, urinary BoE (excluding for nicotine) and eCO levels were significantly (*p* < .05) reduced by medians between 20.9% and 92.1% compared with baseline in all groups either using glo^™^ or iQOS or quitting tobacco use. Between-group comparisons revealed that the reductions in the glo^™^ groups were similar (*p* > .05) to quitting in many cases.

**Conclusions:**

glo^™^ or iQOS use for 5 days reduced exposure to smoke toxicants in a manner comparable to quitting tobacco use. THPs are reduced exposure tobacco products with the potential to be MRTPs.

**Implications:**

This clinical study demonstrates that when smokers switched from smoking combustible cigarettes to using tobacco heating products their exposure to smoke toxicants was significantly decreased. In many cases, this was to the same extent as that seen when they quit smoking completely. This may indicate that these products have the potential to be reduced exposure and/or reduced risk tobacco products when used by smokers whose cigarette consumption is displaced completely.

**Clinical Trial Registrations:**

ISRCTN14301360 and UMIN000024988.

## Introduction

Smoking is a leading cause of numerous human disorders including lung cancer, chronic obstructive pulmonary disease, and cardiovascular disease. Smoking-related disease risk is related to daily cigarette consumption and the number of years since smoking initiation and is principally due to exposure to a number of smoke toxicants transferred from the combustion of tobacco into cigarette smoke.^1–4^ Reducing the health burden of cigarette smoking is a public health priority and has led to the development of a variety of initiatives to reduce toxicant exposure through encouraging abstinence.^5^ More recently, the question has arisen of whether public health gains could be made through the development of new nicotine and tobacco products to support the displacement of combustible cigarette smoking.^6^

Combustible cigarette smoke contains more than 6500 identified chemical constituents,^7^ many of which have been identified as potential contributors to the harmful effects of smoking. The 2001 report by the US Institute of Medicine (IoM) entitled *Clearing the Smoke: the scientific basis for tobacco harm reduction*^2,4^ proposed the development of potential reduced exposure products (PREPs) as a possible means by which to achieve tobacco harm reduction. Although reduced toxicant combustible cigarettes with the properties of a PREP have been described,^8,9^ significant changes in indicators of health were not observed in a long-term clinical study with such products.^10^ Since that time, other forms of tobacco products that heat instead of combust tobacco have been developed.^11,12^ We recently reported a pre-clinical assessment of the glo^™^ tobacco heating product (THP1.0),^13,14^ which electronically heats tobacco to a temperature of 240°C,^12^ and both its yields of machine-measured toxicants and environmental emissions are greatly reduced compared with those from conventional cigarettes.^15,16^ THP1.0 showed no or substantially reduced responses in mutagenic and cytotoxic endpoints relative to cigarettes in both *in vitro* toxicological and contemporary screening assays.^17–19^ Finally, a study with Japanese smokers showed that when they switch to glo^™^ over a 1-week period, they almost fully replaced their combustible cigarette consumption with glo^™^, and their combined daily cigarette and glo^™^ consumable consumption did not increase.^20^ The aim of this current study was to determine whether reductions in machine yields translated into a lowering of toxicant exposure, by measuring biomarkers of exposure (BoE) in a clinical confinement study in Japanese subjects who either continued smoking, used glo^™^/THP1.0 or an in-market comparator THP (iQOS/THS), or abstained completely from tobacco product use, for 5 days.

## Methods

A full description of the study protocol has been published previously.^21^ Brief study details are described here.

### Study Design

This was a randomized, controlled, parallel group open-label clinical confinement study carried out at two sites in Fukuoka, Japan. The study was registered on both the ISRCTN (ISRCTN14301360) and the UMIN (UMIN000024988) registries. Favorable opinion for the study was given by the Hakata Clinic Institutional Review Board, Medical Co LTA, Fukuoka, Japan (reference number 1684CP). The study was conducted in compliance with the ethical principles of the Declaration of Helsinki, Good Clinical Practice (International Conference on Harmonisation E6 Consolidated Guidance, April 1996) and Japanese laws, including those relating to the protection of subjects’ personal data.

### Participants

Healthy male or female smokers were enrolled in this study. During a screening visit, potential participants were assessed for their suitability based on inclusion/exclusion criteria which have been described in full previously.^21^ Main inclusion criteria were current smokers (verified using urinary cotinine [>200 ng/mL] and exhaled breath carbon monoxide [eCO; >10 ppm] tests) who self-reported typically smoking daily at least 10 and a maximum of 30 commercially available cigarettes between the International Organization for Standardization (ISO) tar levels of 6–8 mg/cig; consecutive smoking history of at least 3 years; aged between 23 and 55 years inclusive.

Main exclusion criteria were subjects who did not agree, or whose partners of childbearing potential did not agree, to use effective methods of contraception for the duration of the study from screening to discharge; female subjects who were pregnant or breastfeeding; subjects who had donated ≥400 mL of blood within 12 weeks (males) or 16 weeks (females) prior to admission; subjects who had an acute illness (eg upper respiratory tract infection) requiring treatment within 4 weeks prior to admission; subjects who had regularly used any nicotine or tobacco product other than factory-made filter cigarettes within 14 days of screening; subjects who were self-reported or observed at admission by the clinic staff as non-inhalers of cigarette smoke; and subjects who had any clinically relevant abnormal findings on physical examination, medical history, electrocardiography (ECG), lung function tests, or clinical laboratory panel. Subjects were also excluded if they were planning to quit smoking in the next 12 months. All subjects were informed that they were free to quit smoking and withdraw from the study at any time. Any subject who decided to quit smoking was directed to appropriate stop smoking services.

### Investigational Products

All study cigarettes and THP devices/tobacco consumables were provided by the sponsor free of charge. A single study product was allocated to each group; these products were a 7-mg/cig ISO tar combustible tobacco non-menthol cigarette, glo^™^/THP1.0 with non-menthol Neostiks, a 7-mg/cig ISO tar combustible tobacco menthol cigarette, and glo^™^/THP1.0 with menthol Neostiks. The iQOS/THS product with non-menthol tobacco consumables was also studied as an in-market comparator product. Only those smokers who regularly smoke mentholated cigarettes were randomized to use mentholated products during the study. A further (abstinence) group refrained from using any tobacco or nicotine products after the switch at the end of the baseline period. Aerosol emissions for the THPs used in this study have been published previously.^15,22^ Smoke constituents for the comparator combustible cigarettes manufactured for this study are in Supplementary Table 1.

### Study Procedures

**Figure 1.**
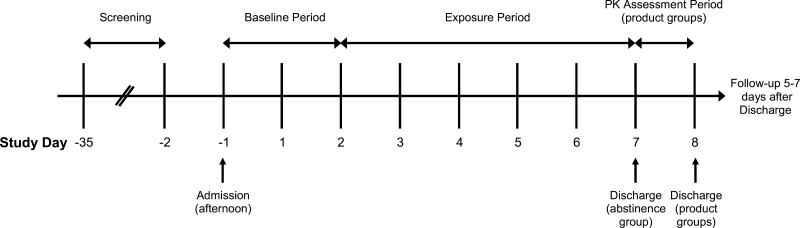
Study design schematic. Subjects completed the baseline period (two consecutive 24-h periods from the evening of day –1 to the evening of day 1, and from the evening of day 1 to the evening of day 2) before moving to the exposure period (five consecutive 24-h periods beginning on the evening of day 2).

A study design schematic is shown in Figure 1. At screening, subjects underwent testing to ensure that they met all inclusion/exclusion criteria. Subjects also completed a tobacco use history questionnaire and the Fagerström Test for Cigarette Dependence.^23^ Subjects who met all inclusion/exclusion criteria were entered into the study. On day –1, subjects entered the clinic, and 24-h urine collection periods began for 2 days, during which time all subjects smoked regular cigarettes. After this period and according to the randomization code, subjects remained smoking regular cigarettes, switched to using a THP, or refrained from using any tobacco products for 5 days. During this period, 24-h urine samples were collected for BoE analysis. On all study days, carbon monoxide in exhaled breath (eCO) was measured using a portable meter (piCO^+^ Smokerlyzer, Bedfont Scientific Ltd, Maidstone, UK). Participant’s consumption of either cigarettes or THP tobacco consumables while in the clinic was limited to no more than 125% of their usual daily cigarette consumption.

At the end of the 5-day exposure period, subjects (other than those in the abstinence group) remained in the clinic for a further day to undergo a nicotine pharmacokinetic assessment with their assigned product. Nicotine pharmacokinetic data from this assessment are not reported in this study.

After the nicotine pharmacokinetic assessment was completed, participants were discharged from the clinic. All subjects were followed up by telephone 5–7 days after discharge to capture whether any subsequent adverse events (AEs) occurred.

### Sample Size Determination

A sample size of 30 subjects per group was determined based on powering the primary objective of within-group comparisons of biomarker levels between baseline and end of study (days 6–7). The calculation was based on the number of pairs required to perform a paired *t* test with 80% power for a decrease in biomarker levels of 40% or more compared with historical biomarker data available for a 7-mg/cig ISO tar conventional cigarette.^9,10^ A sample size of 30 was determined to be adequate based on the biomarker requiring the most pairs to power (eCO, requiring 26 pairs) and allowing for potential attrition. A sample size of 30 was also determined to be able to provide sufficient power for the secondary objective of between-group comparisons, based on a minimum of a 40% reduction in BoE.

### Statistical Methods

Summary statistics and statistical analyses were performed for subjects included in the relevant analysis populations (safety/intent to treat/per protocol). Missing values were not imputed (no missing values were reported), and values below the analytical limit of detection (LOD) or lower limit of quantification (LLOQ) were replaced with half the value of the LOD or LLOQ, respectively. Data analysis was performed using SAS® Version 9.3.

For biomarker data, the mean of the two values taken prior to first randomized product use (ie days –1 to 1 and days 1–2) was used as the baseline value. Mean change from baseline was the mean of all individual subjects’ change from baseline values. Each individual change from baseline was calculated by subtracting the individual subject’s baseline value from the value at the timepoint. Mean percent change from baseline was the mean of all individual subjects’ percent change from baseline values.

The amount excreted for the urinary biomarkers over 24 h and the concentrations for CO in exhaled breath were summarized along with actual changes and percentage changes from baseline at each measurement.

The baseline and days 6–7 values were used to investigate the within-arm changes in biomarkers for each arm separately using a paired *t* test. Only subjects who had both baseline and days 6–7 data were included in the analysis. As a secondary objective, the baseline and days 6–7 values were also used to investigate comparisons between arms, using a mixed ANOVA with fixed terms for site, product use, day, and arm, and a random term for subject. Product use was defined as the number of cigarettes or THP consumables used per day recorded at baseline and days 6–7 by subject. If site was not significant (*p* > .05), it was not included in the model. The LS means difference from baseline was reported for each product separately. For each comparison, the difference in the changes from baseline between two products was also presented along with the 95% confidence intervals.

### Urinary BoE

BoE to selected cigarette smoke constituents in 24-h urine collections were measured throughout the study from days –1 to 7. The study examined the following urinary BoE: total nicotine equivalents (TNeq; nicotine, cotinine, 3-hydroxycotinine, and their glucuronide conjugates); total 4-(methylnitrosamino)-1-(3-pyridyl)-1-butanol (NNAL); total N-nitrosonornicotine (NNN); 3-hydroxypropylmercapturic acid (3-HPMA); 3-hydroxy-1-methylpropylmercapturic acid (HMPMA); S-phenylmercapturic acid (S-PMA); monohydroxybutenyl-mercapturic acid (MHBMA); 2-cyanoethylmercapturic acid (CEMA); 4-aminobiphenyl (4-ABP); o-toluidine (o-Tol); 2-aminonaphthalene (2-AN); 1-hydroxypyrene (1-OHP); 2-hydroxyethylmercapturic acid (HEMA); N-acetyl-S-(2-carbamoylethyl)cysteine (AAMA); and N-acetyl-S-(2-hydroxy-2-carbamoylethyl)cysteine (GAMA).

Laboratory analyses were carried out at Celerion Laboratories (Lincoln, NE, USA) or ABF GmbH (Munich, Germany). Details on the bioanalytical methods have been published previously.^21^

### AEs, Medical History, and Concomitant Medication

Safety assessments included AEs, serious AEs (SAEs), vital signs, ECG, spirometry, clinical chemistry, hematology, urinalysis, physical examinations, and use of concomitant medications. AEs were recorded from the time of signing the informed consent form until the end of the follow-up period after discharge from the clinic. AEs, concomitant diseases, and medical/surgical history were coded using the Medical Dictionary for Regulatory Activities (MedDRA version 19.1).

## Results

### Participant Demographics

Overall 182 participants were enrolled into the study on day –1 and randomized to one of the six study groups. During the baseline period, two participants were withdrawn from the study. One was withdrawn on day 2 due to not meeting the eligibility criteria, and a further was withdrawn due to an AE of nasopharyngitis on day 1. Those withdrawn did not enter the exposure period and were replaced. Thus, 180 participants entered the exposure period and completed the study in accordance with the protocol.

Demographic details of the study participants can be found in Table 1. All participants were Japanese, with a male:female ratio of 1:1. The ratio of male to female subjects was similar across the study groups. The mean age between study groups was similar, ranging from 31 to 35 years of age. The mean body mass index for males and females between the study groups was similar (ranges 21.5–22.2 and 20.7–22.7 kg/m^2^ respectively).

**Table 1.** Demographic Data for Study Participants

	Product							
		Non-menthol cigarette	Non-menthol glo^™^ THP	Menthol cigarette	Menthol glo^™^ THP	iQOS THP	Cessation	Overall
*n*		30	30	30	30	30	30	180
Age (years)	Mean (SD)	32 (8.2)	34 (10.1)	33 (8.6)	31 (7.7)	33 (9.5)	35 (10.0)	33 (9.0)
Sex	Male	15	15	14	16	15	15	90
	Female	15	15	16	14	15	15	90
Weight (males; kg)	Mean (SD)	63.1 (8.5)	63.8 (9.0)	63.9 (6.5)	62.1 (9.0)	65.3 (7.1)	63.9 (6.2)	63.7 (7.7)
Weight (females; kg)	Mean (SD)	54.2 (7.0)	57.7 (8.7)	52.9 (9.4)	52.4 (6.4)	54.7 (8.7)	53.0 (7.3)	54.2 (8.0)
BMI (males)	Mean (SD)	21.9 (3.0)	22.0 (2.7)	22.2 (2.3)	21.5 (2.0)	22.2 (2.2)	21.8 (1.9)	21.9 (2.3)
BMI (females)	Mean (SD)	21.9 (2.8)	22.7 (3.7)	20.7 (2.9)	21.1 (2.0)	22.0 (3.4)	21.1 (2.1)	21.6 (2.9)
FTCD total score	Mean (SD)	5 (2.0)	5 (1.6)	5 (1.7)	4 (2.2)	4 (1.5)	4 (1.6)	4 (1.8)
ISO tar rating^a^ (mg)	Mean (SD)	7 (1.0)	7 (1.0)	8 (0.7)	8 (0.6)	7 (0.9)	7 (1.0)	7 (1.0)
Cigarettes per day^a^	Mean (SD)	17 (5.7)	17 (4.5)	15 (3.9)	15 (4.3)	15 (3.7)	15 (4.4)	16 (4.5)

BMI = body mass index; FTCD = Fagerström Test for Cigarette Dependence; ISO, International Organization for Standardization; THP = tobacco heating product.

^a^ISO tar rating of usual brand cigarette and self-reported cigarette consumption at screening.

All subjects had a smoking history of at least 3 years and smoked between 10 and 30 cigarettes daily (Table 1). Subjects’ chosen cigarette brands were within the ISO tar rating of 6–8 mg/cig, and they had smoked these brands for at least 6 months prior to screening. The number of cigarettes smoked daily was similar between study groups, ranging from a mean of 15 to 17 cigarettes per day (Table 1). The mean ISO tar rating of the cigarettes smoked for all study groups was also similar, ranging from 7 to 8 mg/cig.

Fagerström Test for Cigarette Dependence scores were similar between study groups (Table 1), ranging from 4 to 5 (SDs = 1.5–2.2). Overall, 62 subjects were categorized as having mild scores, 96 were categorized as having moderate scores, and 22 were categorized as having severe scores.

### Tobacco Product Consumption

**Figure 2.**
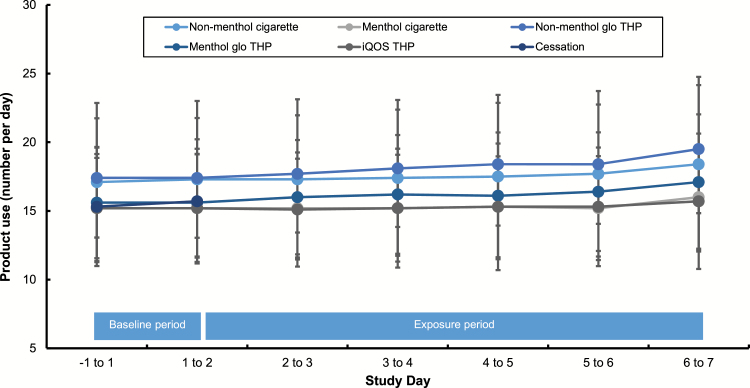
Tobacco product consumption during the study. Data are mean (±SD) numbers of cigarettes smoked/tobacco heating product (THP) consumables used during each study day.

The number of conventional cigarettes/THP consumables used during the baseline and exposure periods can be found in Figure 2. Product use at baseline (days –1 to 1) was similar between study groups, ranging from mean values of 15.2 to 17.4 cig/24 h. A non-significant increase in 24-h product use count was observed for all study groups as the study progressed. The peak product use during the study occurred on days 6–7 for all study groups.

### Biomarkers of Exposure

All 180 subjects had a valid assessment of BoE variables and completed the study. Four subjects had baseline values for o-toluidine that were above the upper limit of quantification and were erroneously high. Data on all days for these subjects were excluded from any summaries and statistical analysis. No data were log-transformed prior to the statistical analyses because the transformation did not markedly change the normality assumptions of the data, and the results of the analyses were still valid based on the raw data.

**Figure 3.**
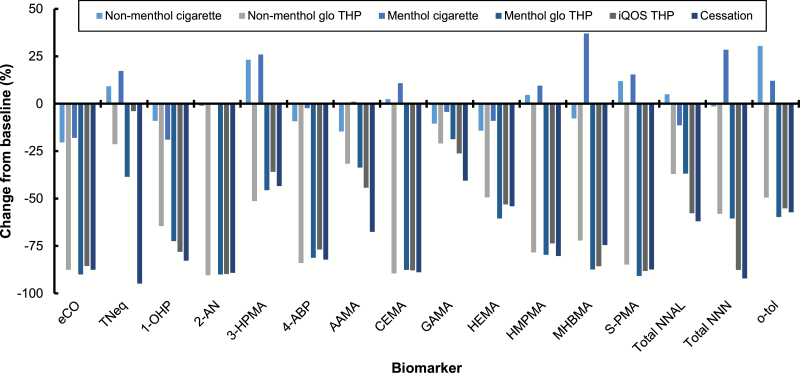
Biomarker of exposure changes between baseline and days 6–7. Data are median values expressed as a percentage of the baseline value. All data, except for eCO, were calculated using biomarker levels from 24-h urine collections at baseline and on days 6–7. eCO levels were calculated from data captured at a single timepoint at baseline and on day 7. *n* = 27–30 in each case. Variability estimates are not shown for clarity; these can be found in Supplementary Table 2 eCO, exhaled carbon monoxide; TNeq, total nicotine equivalents (nicotine, cotinine, 3-hydroxycotinine and their glucuronide conjugates); 1-OHP, 1-hydroxypyrene; 2-AN, 2-aminonaphthalene; 3-HPMA, 3-hydroxypropylmercapturic acid; 4-ABP, 4-aminobiphenyl; AAMA, N-acetyl-S-(2-carbamoylethyl)cysteine; CEMA, 2-cyanoethylmercapturic acid; GAMA, N-acetyl-S-(2-hydroxy-2-carbamoylethyl)cysteine; HEMA, 2-hydroxyethylmercapturic acid; HMPMA, 3-hydroxy-1-methylpropylmercapturic acid; MHBMA, monohydroxybutenyl-mercapturic acid; S-PMA, S-phenylmercapturic acid; NNAL, 4-(methylnitrosamino)-1-(3-pyridyl)-1-butanol (NNAL); NNN, N-nitrosonornicotine; o-tol, o-toluidine.

Changes in BoE levels between baseline and days 6–7 of the exposure period are presented in Figure 3, with statistical data also presented in Supplementary Table 2. All urinary and exhaled BoE assessed following the switch from a conventional cigarette to either menthol or non-menthol variants of glo^™^/THP1.0, the iQOS/THS THP, or to cessation were substantially and significantly decreased from baseline on days 6–7 (*p* < .05) with the exception of TNeq for the iQOS group (*p* = .09). Eight of the 16 BoE assessed were at a level below the analytical limit of quantification in at least one subject on days 6–7 (Supplementary Table 3).

For the majority of the BoE, the magnitudes of the reductions from baseline in the glo^™^ THP groups were similar to those observed in the cessation group. Changes from baseline on days 6–7 for 3-HPMA, HMPMA, S-PMA, MHBMA, CEMA, 4-ABP, o-toluidine, 2-AN, total NNN, and HEMA for both glo^™^ THP groups were not significantly different compared with the cessation group (*p* > .05 in all cases). Similarly, changes from baseline for eCO in the non-menthol glo^™^ THP group, and total NNAL and 1-OHP in the menthol glo^™^ THP group, were not significantly different compared with the cessation group. Finally, although statistically significant decreases from baseline were observed on days 6–7 in both glo^™^ THP variant groups for TNeq, AAMA, and GAMA, the changes from baseline were significantly smaller than those observed in the cessation group (*p* < .05 in each case). The full secondary objectives statistical analysis can be found in Supplementary Table 4.

### Safety

A summary of AEs occurring during the study can be found in Supplementary Tables 5 and 6. No exposure period AEs led to a subject being discontinued from the study.

Overall, 14 exposure period AEs were reported by 10 of the 180 subjects (5.6%). The numbers of exposure period AEs reported and the numbers of subjects reporting exposure period AEs were similar for all study arms. All exposure period AEs were of mild severity, with the exception of one severe AE, which was considered to be an SAE, and one moderate AE. The SAE was a pregnancy detected on discharge from the clinic despite negative pregnancy test results at screening and admission. None of the 14 exposure period AEs were considered to be related to the study product.

The most commonly reported exposure period AE was presyncope. This was reported by 5 of 180 subjects (2.8%): two subjects in the non-menthol cigarette group, one subject in the menthol glo^™^ THP group, and two subjects in the iQOS THP group. All of the presyncope AEs occurred on day 8 of the study during the nicotine pharmacokinetic assessment period, within 8 min of the start of their single-product use. There were 9 AEs reported by 6 of 180 subjects (3.3%) classified as investigations, including a positive urine pregnancy test, 3 AEs of increased alanine aminotransferase, 2 AEs of increased aspartate aminotransferase, 1 AE of glucose urine present, and 2 AEs of increased blood triglycerides. None of the investigation AEs were considered to be related to the study product. There were no clinically significant findings in the vital signs, ECG parameters, and lung function tests data.

## Discussion

Clinical studies examining exposure to toxicants are a key component of an overall assessment of novel tobacco and nicotine products for their impact on human health.^24^ Here, we report data from a randomized, controlled, dual-center open-label study in healthy Japanese smokers conducted to evaluate the effect of switching from a cigarette to menthol and non-menthol variants of the novel THP1.0 on BoE. In addition to the glo^™^ THP, biomarker levels were also investigated in participants who switched to using the in-market comparator THP iQOS/THS, or who completely abstained from any tobacco product use. Overall, use of the study products was safe and well tolerated. The majority of AEs reported were mild in severity, and none were considered related to the study products.

This study assessed 15 urinary BoE to cigarette smoke toxicants and one BoE in exhaled breath. These were selected based on the initial list of priority toxicants proposed by the World Health Organization Study Group on Tobacco Product Regulation^25^ and are of interest due to their potential link to smoking-related health risks. Substantial and statistically significant within-group reductions from baseline for all BoE were observed in subjects who switched from smoking conventional cigarettes to using either glo^™^ THP variant or to using the iQOS THP. For the majority of the biomarkers, the magnitudes of these reductions were not statistically significantly different from those observed in subjects who abstained from using any products after the baseline period. Furthermore, our results are generally consistent with the recent studies on other THP devices in Japan and Poland.^26,27^ While not definitive in terms of demonstrating risk reduction, these toxicant biomarker data are an important component when assessing the potential for changes in risk from tobacco product use.

A secondary analysis of differences between the product use groups and cessation revealed that the urinary levels of the 4-(methylnitrosamino)-1-(3-pyridyl)-1-butanone (NNK) biomarker NNAL, expressed as a percentage of the baseline level, in the menthol glo^™^ THP group were not significantly different from those observed in the cessation group. In contrast, changes in NNAL were significantly different in the non-menthol glo^™^ group, and both menthol and non-menthol cigarette groups compared with the cessation group. The elimination half-life for NNAL is considerably longer (10–18 days) than for the other BoE assessed, and this can lead to carryover when subjects have switched to alternative nicotine products or abstain from tobacco product use.^28^ That NNAL levels were greater in the non-menthol glo^™^ group may be due to slightly higher product consumption than in the iQOS and menthol glo^™^ groups, and residual NNK in the THP tobacco consumable which, though minor compared with that in a combustible cigarette, still gives rise to NNK exposure. Further studies over a period of time longer than that of this 5-day switching study are required to examine the overall impact of THP use on NNK exposure, as well as to the other smoke constituents examined in this study.

The 24-h product use counts were generally similar for all study arms at baseline ranging from mean values of 15.2 to 17.4 cig/24 h. During the exposure period and in all cigarette/THP groups, consumption increased minimally and, generally, by 1–2 cig/tobacco consumables per day. Despite this, participants’ consumption patterns both at baseline and during the exposure period were no greater than their self-reported pre-study consumption. Such escalating product use patterns are typical of confinement studies with tobacco products and have been seen in other studies previously.^10^ Interestingly, an uplift in consumption on the last day on which tobacco products were supplied to study participants was seen in all study groups, including the cessation group at the end of their baseline period. This lends further support to the idea that increases in consumption may be related to the provision of free tobacco products,^29^ with the participants increasing their consumption before this comes to an end.

The increase in non-combustible product consumption as the study progressed did not lead to an increased nicotine consumption, assessed by analyzing urinary excretion of TNeq, as the study progressed. Largely, TNeq levels remained stable or were reduced at the end of the exposure period, compared with those seen at baseline. Where decreases were seen, these were most apparent in the groups using either glo^™^ THP variant. These data suggest that the subjects were adjusting their usage of glo^™^ to achieve their desired levels of nicotine intake.

A number of subjects in all groups had urinary levels of MHBMA which were lower than the level of detection. This could potentially be due to issues with the sample collection and storage prior to assay, or alternatively, it could be due to genotype variations in the population resulting in lower or absent metabolism for this analyte. In support of the latter, there is evidence in the literature to suggest that urinary excretion of MHBMA is lower in Japanese populations with the *GSTT1-null* genotype compared with other ethnic populations.^30^

### Limitations

While this confinement study design provides benefit in facilitating an examination of exposure changes when smokers switch to using THPs in a controlled environment and in the absence of confounding factors such as use of other products and not just the assigned product, it is not without limitation. By its very nature, a confinement study in a clinical environment is not able to determine whether such exposure changes would be seen in a more real-life setting. Thus, one limitation of this study is the lack of insight it gives into exposure reductions that may be observed in an ambulatory setting where other tobacco products are available for use. Secondary to that and further to the discussion on NNK exposure above, the length of this study precludes any conclusions being drawn on long-term changes in exposure to NNK when subjects switch from combusted to heated tobacco. Whilst subject demographics are reported, this study does not explore any potential relationship between changes in biomarker levels and other variables such as age and gender. This study also precludes drawing conclusions on reductions in exposure to smoke constituents other than those for which we examined BoE in this study. Finally, in the absence of conclusive data on potential dose–response relationships or threshold effects, any reductions in BoE cannot be extrapolated to confirm any reductions in health risk.

## Conclusions

The study demonstrated that switching from smoking to using THPs resulted in significant reductions in BoE for selected smoke constituents. For the majority of these biomarkers, the speed and magnitude of the reductions were comparable to those observed during smoking cessation. In this clinical study, the use of the study THPs was safe and well tolerated with a small number of AEs reported that were not attributed to study product use. Together with pre-clinical data on glo^™^/THP1.0 showing reduced emissions and toxicological endpoints relative to cigarettes,^14^ glo^™^ has the potential to be a reduced exposure and/or reduced risk tobacco product when used by smokers whose cigarette consumption is displaced completely.

## Supplementary Material

Supplementary data can be found online at http://www.ntr.oxfordjournals.org.

nty104_suppl_Supplementary_Material

## Funding

The study was sponsored by British American Tobacco (Investments) Limited.

## Declaration of Interests

*NG, MMcE, ACE, OMC, GE, JMcA, JM, CL, and CJP are current employees of British American Tobacco (Investments) Limited (BAT). IMF was an employee of BAT for the duration of the study. NS was a consultant employed by BAT to provide consultancy services for this study. EB, SMcD, EH, AH, SH, LW, and JG are employees of Covance Clinical Research Unit Limited, a contract research organization to provide numerous services to the study.*
