# Supplementary material for: Changes in Biomarkers of Exposure on Switching From a Conventional Cigarette to Tobacco Heating Products: A Randomized, Controlled Study in Healthy Japanese Subjects
Source: Nicotine Tob Res. 2018 Jun 15;21(9):1220–7. doi: 10.1093/ntr/nty104 (PMC6698948; doi:10.1093/ntr/nty104)
Supplement: nty104_suppl_Supplementary_Material [file nty104_suppl_supplementary_material.docx]

**Supplementary Material Table 1. Reference cigarette mainstream** [**smoke**](http://www.sciencedirect.com/topics/pharmacology-toxicology-and-pharmaceutical-science/smoke) **yields for 132 analytes, on a per-cigarette basis, for the study non-menthol and menthol combustible cigarettes.** Yields were measured as described in Forster et al., 2017, using the standard Health Canada intense smoking regime (55 ml puff volume, 2 s puff duration, 30 s puff interval, bell-shaped puff profile and 100% ventilation blocking). BDL, below detectable limits, with limit of detection shown in parentheses. NQ, not quantifiable, with limit of quantification shown in parentheses; SD, standard deviation; n, number of replicates for each of non-menthol and menthol.

| **Parameter** | **Unit** | **Non-menthol cigarette mean** | **Non-menthol cigarette SD** | **Menthol cigarette mean** | **Menthol cigarette SD** | **n** |
| --- | --- | --- | --- | --- | --- | --- |
| 1-aminonaphthalene | ng/cigarette | 16.32 | 0.45 | 20.20 | 0.82 | 5 |
| 2,6-dimethylaniline | ng/cigarette | 5.68 | 0.85 | 6.93 | 0.62 | 5 |
| 2-aminonaphthalene | ng/cigarette | 10.94 | 0.50 | 13.56 | 0.74 | 5 |
| 3-aminobiphenyl | ng/cigarette | 2.98 | 0.24 | 3.47 | 0.33 | 5 |
| 4-aminobiphenyl | ng/cigarette | 2.04 | 0.20 | 2.37 | 0.24 | 5 |
| Benzidine | ng/cigarette | BDL (0.01) | - | BDL (0.01) | - | 5 |
| o-anisidine | ng/cigarette | 3.75 | 0.18 | 4.34 | 0.35 | 5 |
| o-toluidine | ng/cigarette | 73.12 | 2.20 | 87.80 | 6.29 | 5 |
| Ammonia | μg/cigarette | 34.98 | 1.60 | 36.34 | 1.28 | 5 |
| Caffeic Acid | μg/cigarette | BDL (1.19) | - | BDL (1.19) | - | 5 |
| 2,3-Heptanedione | μg/cigarette | BDL (1.17) | - | BDL (1.17) | - | 5 |
| 2,3-Hexanedione | μg/cigarette | BDL (0.954) | - | BDL (0.954) | - | 5 |
| Acetaldehyde | μg/cigarette | 1818.4 | 114.9 | 1800.8 | 144.0 | 5 |
| Acetoin | μg/cigarette | 5.90 | 2.12 | 7.20 | 1.24 | 5 |
| Acetone | μg/cigarette | 537.4 | 53.8 | 530.8 | 18.8 | 5 |
| Acetylpropionyl | μg/cigarette | 28.48 | 6.55 | 30.40 | 1.76 | 5 |
| Acrolein | μg/cigarette | 128.2 | 9.5 | 131.4 | 13.9 | 5 |
| Butyraldehyde | μg/cigarette | 12.96 | 1.35 | 12.88 | 0.94 | 5 |
| Crotonaldehyde | μg/cigarette | 36.84 | 4.10 | 34.76 | 4.32 | 5 |
| Diacetyl | μg/cigarette | 218.8 | 27.4 | 203.2 | 10.3 | 5 |
| Formaldehyde | μg/cigarette | 55.0 | 6.3 | 54.7 | 2.4 | 5 |
| Glycoaldehyde | μg/cigarette | 33.12 | 5.59 | 30.24 | 3.33 | 5 |
| Glyoxal | μg/cigarette | 9.20 | 1.63 | 7.72 | 1.17 | 5 |
| Isobutyraldehyde | μg/cigarette | 40.96 | 3.91 | 41.36 | 2.19 | 5 |
| Methyl ethyl ketone | μg/cigarette | 149.4 | 12.2 | 146.4 | 10.7 | 5 |
| Methyl vinyl ketone | μg/cigarette | 68.12 | 9.74 | 64.76 | 4.51 | 5 |
| Methylglyoxal | μg/cigarette | 31.60 | 4.67 | 31.04 | 1.50 | 5 |
| Propionaldehyde | μg/cigarette | 111.0 | 9.1 | 115.4 | 9.1 | 5 |
| Allyl alcohol | μg/cigarette | 10.52 | 0.92 | 9.79 | 0.73 | 5 |
| EthylCarbamate | ng/cigarette | BDL (6.43) | - | BDL (6.43) | - | 5 |
| Glycerol | mg/cigarette | 0.91 | 0.02 | 0.97 | 0.04 | 5 |
| Total Glycidol | mg/cigarette | NQ (0.006) | - | 0.0060 | 0.0013 | 5 |
| AalphaC | ng/cigarette | 151.0 | 10.6 | 149.4 | 11.1 | 5 |
| Glu-P-1 | ng/cigarette | BDL (0.239) | - | BDL (0.239) | - | 5 |
| Glu-P-2 | ng/cigarette | BDL (0.301) | - | BDL (0.301) | - | 5 |
| IQ | ng/cigarette | 5.56 | 1.89 | 5.99 | 0.90 | 5 |
| MeAalphaC | ng/cigarette | 11.57 | 1.65 | 12.08 | 1.27 | 5 |
| PhIP | ng/cigarette | BDL (0.365) | - | BDL (0.365) | - | 5 |
| Trp-P-1 | ng/cigarette | 3.63 | 0.87 | 3.82 | 0.67 | 5 |
| Trp-P-2 | ng/cigarette | 4.87 | 1.07 | 7.02 | 1.21 | 5 |
| Total hydrogen cyanide | μg/cigarette | 347.2 | 29.7 | 334.8 | 34.9 | 5 |
| Hydrazine | ng/cigarette | BDL (4.08) | - | NQ (12.2) | - | 5 |
| Mercury | ng/cigarette | 3.08 | 0.69 | 3.49 | 0.29 | 5 |
| Arsenic | ng/cigarette | 4.17 | 0.50 | 4.17 | 0.24 | 5 |
| Beryllium | ng/cigarette | BDL (0.936) | - | BDL (0.936) | - | 5 |
| Cadmium | ng/cigarette | 53.62 | 1.11 | 54.94 | 2.74 | 5 |
| Chromium | ng/cigarette | NQ (4.51) | - | NQ (4.51) | - | 5 |
| Cobalt | ng/cigarette | BDL (0.893) | - | BDL (0.893) | - | 5 |
| Copper | ng/cigarette | 36.32 | 1.62 | 35.84 | 1.54 | 5 |
| Iron | ng/cigarette | 41.58 | 13.42 | 35.82 | 3.40 | 5 |
| Lead | ng/cigarette | 21.00 | 0.77 | 20.90 | 1.56 | 5 |
| Nickel | ng/cigarette | NQ (9.49) | - | NQ (9.49) | - | 5 |
| Selenium | ng/cigarette | NQ (2.63) | - | NQ (2.63) | - | 5 |
| Tin | ng/cigarette | BDL (6.04) | - | BDL (6.04) | - | 5 |
| Zinc | ng/cigarette | 288.4 | 49.3 | 272.2 | 35.1 | 5 |
| Nitrogen monoxide | μg/cigarette | 265.2 | 9.8 | 272.4 | 19.6 | 5 |
| Nitrogen oxides | μg/cigarette | 307.2 | 10.4 | 314.2 | 24.3 | 5 |
| Anabasine | ng/cigarette | 1152.8 | 98.9 | 1298.4 | 163.6 | 5 |
| Anatabine | ng/cigarette | 7260.4 | 604.3 | 7613.4 | 530.1 | 5 |
| Cotinine | ng/cigarette | 15305.2 | 1284.0 | 15446.8 | 732.1 | 5 |
| Myosmine | ng/cigarette | 14042.4 | 1768.6 | 15037.8 | 1679.6 | 5 |
| Nicotine-N-oxide | ng/cigarette | 1069.0 | 59.4 | 1015.6 | 223.8 | 5 |
| Nornicotine | ng/cigarette | 19089.2 | 2020.1 | 20864.4 | 1685.3 | 5 |
| beta-nicotyrine | ng/cigarette | 7770.2 | 850.3 | 8408.2 | 312.0 | 5 |
| 2-Nitropropane | ng/cigarette | 33.64 | 3.59 | 41.26 | 4.63 | 5 |
| N-nitrosodi-n-butylamine | ng/cigarette | NQ (1.11) | - | NQ (1.11) | - | 5 |
| N-nitrosodi-n-propylamine | ng/cigarette | BDL (0.15) | - | BDL (0.15) | - | 5 |
| N-nitrosodiethanolamine | ng/cigarette | NQ (0.283) | - | BDL (0.085) | - | 5 |
| N-nitrosodiethylamine | ng/cigarette | BDL (0.617) | - | BDL (0.617) | - | 5 |
| N-nitrosodiisopropylamine | ng/cigarette | BDL (0.546) | - | BDL (0.546) | - | 5 |
| N-nitrosodimethylamine | ng/cigarette | 11.80 | 1.94 | 10.47 | 2.41 | 5 |
| N-nitrosoethylmethylamine | ng/cigarette | BDL (0.509) | - | BDL (0.509) | - | 5 |
| N-nitrosomorpholine | ng/cigarette | BDL (0.55) | - | BDL (0.55) | - | 5 |
| N-nitrosopiperidine | ng/cigarette | BDL (0.172) | - | BDL (0.172) | - | 5 |
| N-nitrosopyrrolidine | ng/cigarette | 6.59 | 1.77 | 8.79 | 2.14 | 5 |
| Benz(j)aceanthrylene | ng/cigarette | 2.33 | 0.27 | 2.92 | 0.59 | 5 |
| Benzo[a]anthracene | ng/cigarette | 26.84 | 2.00 | 34.16 | 3.15 | 5 |
| Benzo[a]pyrene | ng/cigarette | 13.58 | 1.14 | 16.80 | 1.77 | 5 |
| Benzo[c]phenanthrene | ng/cigarette | 9.56 | 0.48 | 11.33 | 1.65 | 5 |
| Chrysene | ng/cigarette | 38.92 | 2.70 | 48.82 | 4.23 | 5 |
| Cyclopenta(c,d)pyrene | ng/cigarette | 10.52 | 0.31 | 13.22 | 1.23 | 5 |
| Indeno(1,2,3-cd)pyrene | ng/cigarette | 4.28 | 0.35 | 5.53 | 0.62 | 5 |
| Naphthalene | ng/cigarette | 1046.0 | 170.8 | 1286.4 | 140.6 | 5 |
| Pyrene | ng/cigarette | 84.58 | 7.32 | 103.70 | 8.80 | 5 |
| 5-methylchrysene | ng/cigarette | 0.59 | 0.13 | 0.68 | 0.21 | 5 |
| Benzo(b)fluoranthene | ng/cigarette | 13.82 | 0.96 | 17.16 | 1.67 | 5 |
| Benzo(k)fluoranthene | ng/cigarette | 4.32 | 0.30 | 5.39 | 0.48 | 5 |
| Dibenz(a,e)pyrene | ng/cigarette | NQ (0.696) | - | NQ (0.696) | - | 5 |
| Dibenz(a,h)anthracene | ng/cigarette | 1.09 | 0.14 | 1.21 | 0.26 | 5 |
| Dibenz(a,h)pyrene | ng/cigarette | BDL (0.236) | - | BDL (0.236) | - | 5 |
| Dibenz(a,i)pyrene | ng/cigarette | 1.35 | 0.25 | 2.04 | 0.43 | 5 |
| Dibenz(a,l)pyrene | ng/cigarette | BDL (0.423) | - | BDL (0.423) | - | 5 |
| Catechol | μg/cigarette | 99.10 | 3.07 | 99.24 | 5.13 | 5 |
| Hydroquinone | μg/cigarette | 107.40 | 3.65 | 114.40 | 5.59 | 5 |
| Phenol | μg/cigarette | 14.86 | 1.11 | 14.90 | 0.39 | 5 |
| Resorcinol | μg/cigarette | 2.32 | 0.35 | 2.48 | 0.31 | 5 |
| m-Cresol | μg/cigarette | 3.29 | 0.19 | 3.42 | 0.17 | 5 |
| o-Cresol | μg/cigarette | 3.58 | 0.27 | 3.74 | 0.23 | 5 |
| p-Cresol | μg/cigarette | 8.55 | 0.53 | 8.82 | 0.35 | 5 |
| Acetamide | μg/cigarette | 10.92 | 0.52 | 11.06 | 1.13 | 5 |
| Acrylamide | μg/cigarette | 4.54 | 0.43 | 4.75 | 0.52 | 5 |
| Benzo(b)furan | μg/cigarette | 0.59 | 0.04 | 0.60 | 0.11 | 5 |
| Nitrobenzene | μg/cigarette | BDL (0.038) | - | BDL (0.038) | - | 5 |
| Pyridine | μg/cigarette | 22.96 | 3.26 | 18.86 | 4.10 | 5 |
| Quinoline | μg/cigarette | 0.41 | 0.03 | 0.45 | 0.03 | 5 |
| Styrene | μg/cigarette | 13.74 | 1.89 | 13.24 | 2.26 | 5 |
| Carbon dioxide | mg/cigarette | 65.84 | 3.61 | 70.24 | 1.68 | 5 |
| Carbon monoxide | mg/cigarette | 24.08 | 1.14 | 26.00 | 1.13 | 5 |
| Diethylene glycol | mg/cigarette | BDL (0.004) | - | BDL (0.004) | - | 5 |
| Ethylene glycol | mg/cigarette | 0.034 | 0.003 | 0.036 | 0.002 | 5 |
| NFDPM | mg/cigarette | 29.82 | 2.67 | 31.16 | 1.52 | 5 |
| Nicotine | mg/cigarette | 1.92 | 0.08 | 2.02 | 0.07 | 5 |
| Propylene glycol | mg/cigarette | 0.59 | 0.03 | 0.64 | 0.03 | 5 |
| Puff count | per cigarette | 8.97 | 0.24 | 9.43 | 0.65 | 5 |
| Total particulate matter | mg/cigarette | 45.94 | 4.34 | 47.38 | 2.23 | 5 |
| Water | mg/cigarette | 14.18 | 1.80 | 14.18 | 0.76 | 5 |
| 4-(N-nitrosomethylamino)-1-(3-pyridyl)-1-butanone (NNK) | ng/cigarette | 93.88 | 1.80 | 94.50 | 11.52 | 5 |
| Nitrosoanabasine (NAB) | ng/cigarette | 14.90 | 1.56 | 17.98 | 2.37 | 5 |
| Nitrosoanatabine (NAT) | ng/cigarette | 158.80 | 9.65 | 161.80 | 7.98 | 5 |
| Nitrosonornicotine (NNN) | ng/cigarette | 89.32 | 4.24 | 95.34 | 6.25 | 5 |
| 1,3-Butadiene | μg/cigarette | 101.10 | 9.82 | 107.22 | 6.65 | 5 |
| Acrylonitrile | μg/cigarette | 18.40 | 3.82 | 15.82 | 1.74 | 5 |
| Benzene | μg/cigarette | 72.40 | 11.99 | 66.92 | 5.61 | 5 |
| Ethylbenzene | μg/cigarette | 12.34 | 2.77 | 11.16 | 0.97 | 5 |
| Ethylene oxide | μg/cigarette | 13.89 | 2.57 | 14.72 | 0.53 | 5 |
| Furan | μg/cigarette | 50.74 | 7.42 | 47.92 | 2.86 | 5 |
| Isoprene | μg/cigarette | 811.40 | 76.43 | 785.60 | 49.55 | 5 |
| Nitromethane | ng/cigarette | 571.2 | 150.0 | 445.2 | 114.4 | 5 |
| Propylene oxide | ng/cigarette | 2137.0 | 261.9 | 2142.2 | 122.7 | 5 |
| Toluene | μg/cigarette | 108.90 | 15.90 | 101.78 | 7.63 | 5 |
| Vinyl acetate | ng/cigarette | 624.8 | 65.6 | 606.6 | 80.5 | 5 |
| Vinyl chloride | ng/cigarette | 66.80 | 3.88 | 73.44 | 6.19 | 5 |

**Supplementary Material Table 2. Statistical analysis of change from baseline to Day 7 in biomarkers of exposure for each product.** Baseline data are the mean of data from Day -1 to 1 and Day 1 to 2 for all biomarkers except eCO. For eCO, data are the means of the two measurements made on Day 1 and Day 2. ppm, parts per million; CI, confidence intervals; eCO, exhaled carbon monoxide; TNeq, total nicotine equivalents (nicotine, cotinine, 3-hydroxycotinine and their glucuronide conjugates); 1-OHP, 1-hydroxypyrene; 2-AN, 2-aminonaphthalene; 3-HPMA, 3-hydroxypropylmercapturic acid; 4-ABP, 4-aminobiphenyl; AAMA, N-acetyl-S-(2-carbamoylethyl)cysteine; CEMA, 2-cyanoethylmercapturic acid; GAMA, N‑acetyl-S-(2-hydroxy-2-carbamoylethyl)cysteine; HEMA, 2-hydroxyethylmercapturic acid; HMPMA, 3-hydroxy-1-methylpropylmercapturic acid; MHBMA, monohydroxybutenyl-mercapturic acid ; S-PMA, S-phenylmercapturic acid; total NNAL, 4-(methylnitrosamino)-1-(3-pyridyl)-1-butanol (NNAL); total NNN, N-nitrosonornicotine; o-tol, o-toluidine.

| **Biomarker (units)** | **Product** | **N** | **Baseline mean** | **Day 7 mean** | **Mean difference (Day 7 - baseline)** | **95% CI of the difference** | ***p* value** |
| --- | --- | --- | --- | --- | --- | --- | --- |
| **eCO (ppm)** | Non-menthol cigarette | 30 | 24.48 | 20.30 | -4.18 | (-7.42, -0.94) | 0.013 |
|  | Non-menthol glo^TM^ THP | 30 | 26.67 | 3.40 | -23.27 | (-27.02, -19.51) | <0.001 |
|  | Menthol cigarette | 30 | 24.55 | 20.07 | -4.48 | (-6.07, -2.89) | <0.001 |
|  | Menthol glo^TM^ THP | 30 | 27.00 | 2.80 | -24.20 | (-28.18, -20.22) | <0.001 |
|  | iQOS THP | 30 | 23.18 | 3.40 | -19.78 | (-22.84, -16.73) | <0.001 |
|  | Cessation | 30 | 22.72 | 3.03 | -19.68 | (-22.37, -17.00) | <0.001 |
| **TNeq (mg/24h)** | Non-menthol cigarette | 30 | 7.53 | 8.33 | 0.80 | (-0.03, 1.64) | 0.059 |
|  | Non-menthol glo^TM^ THP | 30 | 8.17 | 6.15 | -2.02 | (-3.03, -1.01) | <0.001 |
|  | Menthol cigarette | 30 | 8.25 | 9.77 | 1.51 | (0.87, 2.16) | <0.001 |
|  | Menthol glo^TM^ THP | 30 | 9.29 | 5.75 | -3.54 | (-4.33, -2.76) | <0.001 |
|  | iQOS THP | 30 | 8.20 | 7.58 | -0.63 | (-1.36, 0.10) | 0.090 |
|  | Cessation | 30 | 8.55 | 0.48 | -8.07 | (-9.22, -6.92) | <0.001 |
| **1-OHP (ng/24h)** | Non-menthol cigarette | 30 | 186.99 | 172.86 | -14.13 | (-27.40, -0.87) | 0.038 |
|  | Non-menthol glo^TM^ THP | 30 | 211.33 | 75.58 | -135.74 | (-158.56, -112.93) | <0.001 |
|  | Menthol cigarette | 30 | 237.81 | 195.19 | -42.61 | (-55.98, -29.25) | <0.001 |
|  | Menthol glo^TM^ THP | 30 | 239.46 | 63.46 | -176.00 | (-207.07, -144.93) | <0.001 |
|  | iQOS THP | 30 | 236.52 | 50.18 | -186.34 | (-213.75, -158.92) | <0.001 |
|  | Cessation | 30 | 215.97 | 39.60 | -176.37 | (-200.62, -152.12) | <0.001 |
| **2-AN (ng/24h)** | Non-menthol cigarette | 30 | 17.79 | 17.80 | 0.01 | (-1.57, 1.59) | 0.987 |
|  | Non-menthol glo^TM^ THP | 30 | 18.57 | 1.74 | -16.83 | (-19.52, -14.14) | <0.001 |
|  | Menthol cigarette | 30 | 17.62 | 17.65 | 0.03 | (-1.11, 1.17) | 0.955 |
|  | Menthol glo^TM^ THP | 30 | 19.58 | 1.92 | -17.66 | (-20.91, -14.42) | <0.001 |
|  | iQOS THP | 30 | 17.11 | 1.72 | -15.39 | (-18.52, -12.27) | <0.001 |
|  | Cessation | 30 | 17.61 | 1.79 | -15.82 | (-18.12, -13.52) | <0.001 |
| **3-HPMA (μg/24h)** | Non-menthol cigarette | 30 | 1177.15 | 1448.93 | 271.78 | (139.64, 403.91) | <0.001 |
|  | Non-menthol glo^TM^ THP | 30 | 1208.79 | 568.66 | -640.13 | (-824.77, -455.49) | <0.001 |
|  | Menthol cigarette | 30 | 1136.31 | 1422.37 | 286.05 | (212.25, 359.86) | <0.001 |
|  | Menthol glo^TM^ THP | 30 | 1281.90 | 656.99 | -624.91 | (-799.23, -450.59) | <0.001 |
|  | iQOS THP | 30 | 1021.58 | 639.21 | -382.37 | (-504.80, -259.93) | <0.001 |
|  | Cessation | 30 | 1090.83 | 582.62 | -508.21 | (-668.52, -347.91) | <0.001 |
| **4-ABP (ng/24h)** | Non-menthol cigarette | 30 | 11.79 | 10.86 | -0.93 | (-1.76, -0.09) | 0.031 |
|  | Non-menthol glo^TM^ THP | 30 | 12.61 | 2.45 | -10.17 | (-11.73, -8.60) | <0.001 |
|  | Menthol cigarette | 30 | 10.89 | 10.44 | -0.46 | (-1.06, 0.14) | 0.130 |
|  | Menthol glo^TM^ THP | 30 | 12.76 | 2.31 | -10.45 | (-12.73, -8.18) | <0.001 |
|  | iQOS THP | 30 | 10.35 | 2.25 | -8.10 | (-9.52, -6.67) | <0.001 |
|  | Cessation | 30 | 11.13 | 2.21 | -8.93 | (-10.31, -7.54) | <0.001 |
| **AAMA (μg/24h)** | Non-menthol cigarette | 30 | 129.62 | 111.65 | -17.97 | (-28.19, -7.76) | 0.001 |
|  | Non-menthol glo^TM^ THP | 30 | 133.92 | 91.75 | -42.18 | (-55.32, -29.04) | <0.001 |
|  | Menthol cigarette | 30 | 115.05 | 114.96 | -0.09 | (-7.85, 7.67) | 0.981 |
|  | Menthol glo^TM^ THP | 30 | 132.81 | 88.82 | -43.99 | (-52.30, -35.68) | <0.001 |
|  | iQOS THP | 30 | 117.01 | 65.76 | -51.26 | (-59.81, -42.70) | <0.001 |
|  | Cessation | 30 | 124.58 | 40.60 | -83.97 | (-94.94, -73.01) | <0.001 |
| **CEMA (μg/24h)** | Non-menthol cigarette | 30 | 153.78 | 159.04 | 5.26 | (-8.82, 19.34) | 0.451 |
|  | Non-menthol glo^TM^ THP | 30 | 165.75 | 17.84 | -147.91 | (-173.47, -122.36) | <0.001 |
|  | Menthol cigarette | 30 | 159.65 | 165.62 | 5.96 | (-7.54, 19.46) | 0.374 |
|  | Menthol glo^TM^ THP | 30 | 172.51 | 21.03 | -151.48 | (-175.62, -127.35) | <0.001 |
|  | iQOS THP | 30 | 128.96 | 16.54 | -112.43 | (-133.33, -91.52) | <0.001 |
|  | Cessation | 30 | 140.93 | 15.91 | -125.02 | (-140.28, -109.77) | <0.001 |
| **GAMA (μg/24h)** | Non-menthol cigarette | 30 | 19.27 | 17.24 | -2.03 | (-3.66, -0.40) | 0.016 |
|  | Non-menthol glo^TM^ THP | 30 | 20.34 | 15.68 | -4.66 | (-6.96, -2.36) | <0.001 |
|  | Menthol cigarette | 30 | 17.67 | 16.40 | -1.26 | (-2.88, 0.36) | 0.122 |
|  | Menthol glo^TM^ THP | 30 | 19.32 | 15.36 | -3.95 | (-5.82, -2.09) | <0.001 |
|  | iQOS THP | 30 | 19.05 | 13.75 | -5.30 | (-7.02, -3.58) | <0.001 |
|  | Cessation | 30 | 17.95 | 10.79 | -7.16 | (-8.77, -5.56) | <0.001 |
| **HEMA (μg/24h)** | Non-menthol cigarette | 30 | 6.27 | 5.08 | -1.20 | (-2.04, -0.35) | 0.007 |
|  | Non-menthol glo^TM^ THP | 30 | 5.65 | 2.46 | -3.19 | (-4.37, -2.01) | <0.001 |
|  | Menthol cigarette | 30 | 8.60 | 7.13 | -1.48 | (-2.85, -0.10) | 0.036 |
|  | Menthol glo^TM^ THP | 30 | 7.23 | 2.84 | -4.39 | (-5.61, -3.16) | <0.001 |
|  | iQOS THP | 30 | 6.44 | 2.60 | -3.84 | (-5.06, -2.61) | <0.001 |
|  | Cessation | 30 | 6.87 | 2.55 | -4.32 | (-6.03, -2.61) | <0.001 |
| **HMPMA (μg/24h)** | Non-menthol cigarette | 30 | 356.38 | 385.50 | 29.11 | (-3.89, 62.12) | 0.082 |
|  | Non-menthol glo^TM^ THP | 30 | 372.98 | 79.00 | -293.99 | (-355.77, -230.20) | <0.001 |
|  | Menthol cigarette | 30 | 342.03 | 362.45 | 20.43 | (-12.06, 52.91) | 0.209 |
|  | Menthol glo^TM^ THP | 30 | 383.91 | 73.23 | -310.68 | (-368.56, -252.79) | <0.001 |
|  | iQOS THP | 30 | 333.61 | 79.63 | -253.98 | (-309.32, -198.64) | <0.001 |
|  | Cessation | 30 | 332.39 | 67.63 | -264.76 | (-303.09, -226.43) | <0.001 |
| **MHBMA (ng/24h)** | Non-menthol cigarette | 30 | 750.78 | 770.64 | 19.86 | (-123.66, 163.38) | 0.779 |
|  | Non-menthol glo^TM^ THP | 30 | 574.54 | 49.87 | -524.67 | (-769.55, -279.79) | <0.001 |
|  | Menthol cigarette | 30 | 942.61 | 1010.18 | 67.57 | (-151.04, 286.17) | 0.532 |
|  | Menthol glo^TM^ THP | 30 | 935.36 | 98.40 | -836.96 | (-1157.18, -516.57) | <0.001 |
|  | iQOS THP | 30 | 754.16 | 118.38 | -635.78 | (-927.54, -344.03) | <0.001 |
|  | Cessation | 30 | 720.65 | 107.48 | -613.17 | (-916.08, -310.26) | <0.001 |
| **S-PMA (μg/24h)** | Non-menthol cigarette | 30 | 2.09 | 2.25 | 0.17 | (-0.05, 0.38) | 0.123 |
|  | Non-menthol glo^TM^ THP | 30 | 1.84 | 0.20 | -1.63 | (-2.17, -1.10) | <0.001 |
|  | Menthol cigarette | 30 | 2.68 | 2.81 | 0.13 | (-0.21, 0.48) | 0.438 |
|  | Menthol glo^TM^ THP | 30 | 2.66 | 0.20 | -2.46 | (-3.26, -1.65) | <0.001 |
|  | iQOS THP | 30 | 1.86 | 0.19 | -1.67 | (-2.12, -1.23) | <0.001 |
|  | Cessation | 30 | 1.97 | 0.18 | -1.79 | (-2.34, -1.24) | <0.001 |
| **Total NNAL (ng/24h)** | Non-menthol cigarette | 30 | 188.43 | 197.85 | 9.42 | (-10.29, 29.13) | 0.337 |
|  | Non-menthol glo^TM^ THP | 30 | 198.10 | 128.63 | -69.47 | (-90.09, -48.85) | <0.001 |
|  | Menthol cigarette | 30 | 188.59 | 167.02 | -21.57 | (-38.70, -4.44) | 0.015 |
|  | Menthol glo^TM^ THP | 30 | 237.06 | 149.38 | -87.68 | (-119.14, -56.22) | <0.001 |
|  | iQOS THP | 30 | 174.33 | 80.35 | -93.98 | (-115.46, -72.51) | <0.001 |
|  | Cessation | 30 | 178.18 | 72.39 | -105.79 | (-133.04, -78.54) | <0.001 |
| **Total NNN (ng/24h)** | Non-menthol cigarette | 30 | 15.32 | 15.36 | 0.04 | (-2.75, 2.83) | 0.976 |
|  | Non-menthol glo^TM^ THP | 30 | 11.55 | 5.85 | -5.71 | (-7.46, -3.95) | <0.001 |
|  | Menthol cigarette | 30 | 8.04 | 9.62 | 1.58 | (0.29, 2.87) | 0.018 |
|  | Menthol glo^TM^ THP | 30 | 11.58 | 5.57 | -6.00 | (-9.28, -2.72) | <0.001 |
|  | iQOS THP | 30 | 9.13 | 1.06 | -8.07 | (-10.77, -5.38) | <0.001 |
|  | Cessation | 30 | 8.76 | 0.81 | -7.95 | (-9.73, -6.17) | <0.001 |
| **o-tol (ng/24h)** | Non-menthol cigarette | 27 | 129.80 | 153.21 | 23.41 | (-2.97, 49.80) | 0.080 |
|  | Non-menthol glo^TM^ THP | 29 | 114.26 | 58.52 | -55.74 | (-70.84, -40.64) | <0.001 |
|  | Menthol cigarette | 30 | 114.96 | 119.04 | 4.08 | (-19.29, 27.46) | 0.724 |
|  | Menthol glo^TM^ THP | 30 | 106.89 | 39.39 | -67.50 | (-81.25, -53.74) | <0.001 |
|  | iQOS THP | 30 | 107.96 | 54.81 | -53.16 | (-76.33, -29.99) | <0.001 |
|  | Cessation | 30 | 103.18 | 41.55 | -61.62 | (-71.27, -51.98) | <0.001 |

Supplementary Material Table 3. Frequency of Day 6 to 7 values below lower limit of quantification. 30 Day 6 to 7 values were reported per biomarker, per product group, excluding TNeq which consists of nicotine + 5 metabolites, equating to 180 Day 6 to 7 values per product group. ( ), percentage of values below lower limit of quantification; eCO, exhaled carbon monoxide; TNeq, total nicotine equivalents (nicotine, cotinine, 3-hydroxycotinine and their glucuronide conjugates); 1-OHP, 1-hydroxypyrene; 2-AN, 2-aminonaphthalene; 3-HPMA, 3-hydroxypropylmercapturic acid; 4-ABP, 4-aminobiphenyl; AAMA, N-acetyl-S-(2-carbamoylethyl)cysteine; CEMA, 2-cyanoethylmercapturic acid; GAMA, N acetyl-S-(2-hydroxy-2-carbamoylethyl)cysteine; HEMA, 2-hydroxyethylmercapturic acid; HMPMA, 3-hydroxy-1-methylpropylmercapturic acid; MHBMA, monohydroxybutenyl-mercapturic acid ; S-PMA, S-phenylmercapturic acid; NNAL, 4-(methylnitrosamino)-1-(3-pyridyl)-1-butanol (NNAL); NNN, N-nitrosonornicotine; o-tol, o-toluidine.

|  | **Product Group** | | | | | |
| --- | --- | --- | --- | --- | --- | --- |
| **Biomarker** | **Non-menthol cigarette** | **Non-menthol glo^TM^ THP** | **Menthol cigarette** | **Menthol glo^TM^ THP** | **iQOS THP** | **Cessation** |
| **eCO** | 0 | 0 | 0 | 0 | 0 | 0 |
| **TNeq** | 11 (6%) | 8 (4%) | 7 (4%) | 12 (7%) | 11 (6%) | 150 (83%) |
| **1-OHP** | 0 | 0 | 0 | 0 | 0 | 1 (3%) |
| **2-AN** | 0 | 28 (94%) | 0 | 23 (77%) | 25 (83%) | 26 (87%) |
| **3-HPMA** | 0 | 0 | 0 | 0 | 0 | 0 |
| **4-ABP** | 0 | 14 (47%) | 0 | 15 (50%) | 13 (43%) | 15 (50%) |
| **AAMA** | 0 | 0 | 0 | 0 | 0 | 1 (3%) |
| **CEMA** | 0 | 0 | 0 | 0 | 0 | 0 |
| **GAMA** | 0 | 0 | 0 | 0 | 0 | 0 |
| **HEMA** | 0 | 0 | 0 | 0 | 0 | 0 |
| **HMPMA** | 0 | 0 | 0 | 0 | 0 | 0 |
| **MHBMA** | 13 (43%) | 29 (97%) | 4 (13%) | 24 (80%) | 24 (80%) | 25 (83%) |
| **S-PMA** | 0 | 0 | 0 | 0 | 0 | 0 |
| **Total NNAL** | 0 | 0 | 0 | 0 | 2 (7%) | 0 |
| **Total NNN** | 1 (3%) | 12 (40%) | 1 (3%) | 15 (50%) | 26 (87%) | 29 (97%) |
| **o-tol** | 0 | 0 | 0 | 0 | 0 | 0 |

**Supplementary Material Table 4. Statistical analysis of biomarker of exposure data, examining differences between study groups (secondary objective).** LS mean, least squares mean. ppm, parts per million; CI, confidence intervals; eCO, exhaled carbon monoxide; TNeq, total nicotine equivalents (nicotine, cotinine, 3-hydroxycotinine and their glucuronide conjugates); 1-OHP, 1-hydroxypyrene; 2-AN, 2-aminonaphthalene; 3-HPMA, 3-hydroxypropylmercapturic acid; 4-ABP, 4-aminobiphenyl; AAMA, N-acetyl-S-(2-carbamoylethyl)cysteine; CEMA, 2-cyanoethylmercapturic acid; GAMA, N‑acetyl-S-(2-hydroxy-2-carbamoylethyl)cysteine; HEMA, 2-hydroxyethylmercapturic acid; HMPMA, 3-hydroxy-1-methylpropylmercapturic acid; MHBMA, monohydroxybutenyl-mercapturic acid ; S-PMA, S-phenylmercapturic acid; NNAL, 4-(methylnitrosamino)-1-(3-pyridyl)-1-butanol (NNAL); NNN, N-nitrosonornicotine; o-tol, o-toluidine.

| **Biomarker (units)** | **Product** | **n** | **LS mean** | **Comparison**  **(Test-Reference)** | **Mean difference**  **(Test-Reference)** | **95% CI of the difference** | ***p* value** |
| --- | --- | --- | --- | --- | --- | --- | --- |
| **eCO (ppm)** | Non-menthol cigarette (A) | 30 | -4.18 | B-A | -19.08 | (-23.38, -14.78) | <.001 |
|  | Non-menthol glo^TM^ THP (B) | 30 | -23.27 | B-F | -3.58 | (-7.88, 0.72) | 0.102 |
|  | Menthol cigarette (C) | 30 | -4.48 | D-C | -19.72 | (-24.02, -15.42) | <.001 |
|  | Menthol glo^TM^ THP (D) | 30 | -24.20 | D-F | -4.52 | (-8.82, -0.22) | 0.040 |
|  | iQOS THP (E) | 30 | -19.78 | A-F | 15.50 | (11.20, 19.80) | <.001 |
|  | Cessation (F) | 30 | -19.68 | C-F | 15.20 | (10.90, 19.50) | <.001 |
|  |  |  |  | E-A | -15.60 | (-19.90, -11.30) | <.001 |
| **TNeq (mg/24h)** | Non-menthol cigarette (A) | 30 | 0.80 | B-A | -2.82 | (-4.02, -1.63) | <.001 |
|  | Non-menthol glo^TM^ THP (B) | 30 | -2.02 | B-F | 6.05 | (4.85, 7.25) | <.001 |
|  | Menthol cigarette (C) | 30 | 1.51 | D-C | -5.06 | (-6.26, -3.86) | <.001 |
|  | Menthol glo^TM^ THP (D) | 30 | -3.54 | D-F | 4.53 | (3.33, 5.72) | <.001 |
|  | iQOS THP (E) | 30 | -0.63 | A-F | 8.87 | (7.67, 10.07) | <.001 |
|  | Cessation (F) | 30 | -8.07 | C-F | 9.58 | (8.39, 10.78) | <.001 |
|  |  |  |  | E-A | -1.43 | (-2.63, -0.23) | 0.020 |
| **1-OHP (ng/24h)** | Non-menthol cigarette (A) | 30 | -14.13 | B-A | -121.61 | (-153.03, -90.19) | <.001 |
|  | Non-menthol glo^TM^ THP (B) | 30 | -135.47 | B-F | 40.63 | (9.21, 72.05) | 0.012 |
|  | Menthol cigarette (C) | 30 | -42.61 | D-C | -133.39 | (-164.81, -101.97) | <.001 |
|  | Menthol glo^TM^ THP (D) | 30 | -176.00 | D-F | 0.37 | (-31.05, 31.79) | 0.982 |
|  | iQOS THP (E) | 30 | -186.34 | A-F | 162.24 | (130.82, 193.66) | <.001 |
|  | Cessation (F) | 30 | -176.37 | C-F | 133.76 | (102.34, 165.18) | <.001 |
|  |  |  |  | E-A | -172.21 | (-203.63, -140.79) | <.001 |
| **2-AN (ng/24h)** | Non-menthol cigarette (A) | 30 | -0.01 | B-A | -16.84 | (-20.21, -13.47) | <.001 |
|  | Non-menthol glo^TM^ THP (B) | 30 | -16.83 | B-F | -1.01 | (-4.38, 2.37) | 0.556 |
|  | Menthol cigarette (C) | 30 | 0.03 | D-C | -17.70 | (-21.07, -14.32) | <.001 |
|  | Menthol glo^TM^ THP (D) | 30 | -17.66 | D-F | -1.84 | (-5.22, 1.53) | 0.282 |
|  | iQOS THP (E) | 30 | -15.39 | A-F | 15.83 | (12.46, 19.21) | <.001 |
|  | Cessation (F) | 30 | -15.82 | C-F | 15.85 | (12.48, 19.23) | <.001 |
|  |  |  |  | E-A | -15.41 | (-18.78, -12.03) | <.001 |
| **3-HPMA (μg/24h)** | Non-menthol cigarette (A) | 30 | 271.78 | B-A | -911.91 | (-1111.30, -712.52) | <.001 |
|  | Non-menthol glo^TM^ THP (B) | 30 | -640.13 | B-F | -131.92 | (-331.31, 67.47) | 0.193 |
|  | Menthol cigarette (C) | 30 | 286.05 | D-C | -910.96 | (-1110.35, -711.51) | <.001 |
|  | Menthol glo^TM^ THP (D) | 30 | -624.91 | D-F | -116.69 | (-316.08, 82.70) | 0.250 |
|  | iQOS THP (E) | 30 | -382.37 | A-F | 779.99 | (580.60, 979.38) | <.001 |
|  | Cessation (F) | 30 | -508.21 | C-F | 794.27 | (594.88, 993.66) | <.001 |
|  |  |  |  | E-A | -654.14 | (-853.53, -454.75) | <.001 |
| **4-ABP (ng/24h)** | Non-menthol cigarette (A) | 30 | -0.93 | B-A | -9.24 | (-11.22, -7.26) | <.001 |
|  | Non-menthol glo^TM^ THP (B) | 30 | -10.17 | B-F | -1.24 | (-3.22, 0.74) | 0.218 |
|  | Menthol cigarette (C) | 30 | -0.46 | D-C | -9.99 | (-11.97, -8.01) | <.001 |
|  | Menthol glo^TM^ THP (D) | 30 | -10.45 | D-F | -1.53 | (-3.51, 0.45) | 0.130 |
|  | iQOS THP (E) | 30 | -8.10 | A-F | 8.00 | (6.02, 9.98) | <.001 |
|  | Cessation (F) | 30 | -8.93 | C-F | 8.47 | (6.49, 10.45) | <.001 |
|  |  |  |  | E-A | -7.17 | (-9.15, -5.19) | <.001 |
| **AAMA (μg/24h)** | Non-menthol cigarette (A) | 30 | -17.97 | B-A | -24.21 | (-37.85, -10.56) | <.001 |
|  | Non-menthol glo^TM^ THP (B) | 30 | -42.18 | B-F | 41.80 | (28.15, 55.44) | <.001 |
|  | Menthol cigarette (C) | 30 | -0.09 | D-C | -43.90 | (-57.54, -30.26) | <.001 |
|  | Menthol glo^TM^ THP (D) | 30 | -43.99 | D-F | 39.99 | (26.34, 53.63) | <.001 |
|  | iQOS THP (E) | 30 | -51.26 | A-F | 66.00 | (52.36, 79.65) | <.001 |
|  | Cessation (F) | 30 | -83.97 | C-F | 83.89 | (70.24, 97.53) | <.001 |
|  |  |  |  | E-A | -33.28 | (-46.93, -19.64) | <.001 |
| **CEMA (μg/24h)** | Non-menthol cigarette (A) | 30 | 5.26 | B-A | -153.17 | (-179.81, -126.54) | <.001 |
|  | Non-menthol glo^TM^ THP (B) | 30 | -147.91 | B-F | -22.89 | (-49.53, 3.75) | 0.092 |
|  | Menthol cigarette (C) | 30 | 5.96 | D-C | -157.45 | (-184.08, -130.81) | <.001 |
|  | Menthol glo^TM^ THP (D) | 30 | -151.48 | D-F | -26.46 | (-53.10, 0.18) | 0.052 |
|  | iQOS THP (E) | 30 | -112.43 | A-F | 130.28 | (103.65, 156.92) | <.001 |
|  | Cessation (F) | 30 | -125.02 | C-F | 130.99 | (104.35, 157.62) | <.001 |
|  |  |  |  | E-A | -117.69 | (-144.33, -91.05) | <.001 |
| **GAMA (μg/24h)** | Non-menthol cigarette (A) | 30 | -2.03 | B-A | -2.63 | (-5.10, -0.17) | 0.036 |
|  | Non-menthol glo^TM^ THP (B) | 30 | -4.66 | B-F | 2.50 | (0.04, 4.97) | 0.047 |
|  | Menthol cigarette (C) | 30 | -1.26 | D-C | -2.69 | (-5.16, -0.23) | 0.033 |
|  | Menthol glo^TM^ THP (D) | 30 | -3.95 | D-F | 3.21 | (0.75, 5.68) | 0.011 |
|  | iQOS THP (E) | 30 | -5.30 | A-F | 5.13 | (2.67, 7.60) | <.001 |
|  | Cessation (F) | 30 | -7.16 | C-F | 5.90 | (3.44, 8.37) | <.001 |
|  |  |  |  | E-A | -3.27 | (-5.74, -0.81) | 0.010 |
| **HEMA (μg/24h)** | Non-menthol cigarette (A) | 30 | -1.20 | B-A | -1.99 | (-3.75, -0.24) | 0.026 |
|  | Non-menthol glo^TM^ THP (B) | 30 | -3.19 | B-F | 1.13 | (-0.63, 2.89) | 0.206 |
|  | Menthol cigarette (C) | 30 | -1.48 | D-C | -2.91 | (-4.67, -1.16) | <.001 |
|  | Menthol glo^TM^ THP (D) | 30 | -4.39 | D-F | -0.07 | (-1.82, 1.69) | 0.939 |
|  | iQOS THP (E) | 30 | -3.84 | A-F | 3.12 | (1.37, 4.88) | <.001 |
|  | Cessation (F) | 30 | -4.32 | C-F | 2.84 | (1.09, 4.60) | 0.002 |
|  |  |  |  | E-A | -2.64 | (-4.40, -0.89) | 0.003 |
| **HMPMA (μg/24h)** | Non-menthol cigarette (A) | 30 | 29.11 | B-A | -323.10 | (-389.25, -256.95) | <.001 |
|  | Non-menthol glo^TM^ THP (B) | 30 | -293.99 | B-F | -29.23 | (-95.38, 36.92) | 0.384 |
|  | Menthol cigarette (C) | 30 | 20.43 | D-C | -331.10 | (-397.25, -264.95) | <.001 |
|  | Menthol glo^TM^ THP (D) | 30 | -310.68 | D-F | -45.92 | (-112.07, 20.23) | 0.172 |
|  | iQOS THP (E) | 30 | -253.98 | A-F | 293.87 | (227,27, 360.02) | <.001 |
|  | Cessation (F) | 30 | -264.76 | C-F | 285.18 | (219.04, 351.33) | <.001 |
|  |  |  |  | E-A | -283.10 | (-349.25, -216.95) | <.001 |
| **MHBMA (ng/24h)** | Non-menthol cigarette (A) | 30 | 19.86 | B-A | -544.53 | (-900.31, -188.75) | 0.003 |
|  | Non-menthol glo^TM^ THP (B) | 30 | -524.67 | B-F | 88.50 | (-267.28, 444.28) | 0.624 |
|  | Menthol cigarette (C) | 30 | 67.57 | D-C | -904.53 | (-1260.31, -548.75) | <.001 |
|  | Menthol glo^TM^ THP (D) | 30 | -836.96 | D-F | -223.79 | (-579.57, 131.99) | 0.216 |
|  | iQOS THP (E) | 30 | -635.78 | A-F | 633.03 | (277.25, 988.81) | <.001 |
|  | Cessation (F) | 30 | -613.17 | C-F | 680.73 | (324.95, 1036.51) | <.001 |
|  |  |  |  | E-A | -655.64 | (-1011.42, -299.86) | <.001 |
| **S-PMA (μg/24h)** | Non-menthol cigarette (A) | 30 | 0.17 | B-A | -1.80 | (-2.51, -1.10) | <.001 |
|  | Non-menthol glo^TM^ THP (B) | 30 | -1.63 | B-F | 0.15 | (-0.55, 0.86) | 0.667 |
|  | Menthol cigarette (C) | 30 | 0.13 | D-C | -2.59 | (-3.30, -1.89) | <.001 |
|  | Menthol glo^TM^ THP (D) | 30 | -2.46 | D-F | -0.67 | (-1.37, 0.04) | 0.063 |
|  | iQOS THP (E) | 30 | -1.67 | A-F | 1.96 | (1.25, 2.66) | <.001 |
|  | Cessation (F) | 30 | -1.79 | C-F | 1.92 | (1.22, 2.63) | <.001 |
|  |  |  |  | E-A | -1.84 | (-2.55, -1.13) | <.001 |
| **Total NNAL (ng/24h)** | Non-menthol cigarette (A) | 30 | 9.42 | B-A | -78.89 | (-110.89, -46.88) | <.001 |
|  | Non-menthol glo^TM^ THP (B) | 30 | -69.47 | B-F | 36.32 | (4.31, 68.33) | 0.026 |
|  | Menthol cigarette (C) | 30 | -21.57 | D-C | -66.11 | (-98.12, -34.10) | <.001 |
|  | Menthol glo^TM^ THP (D) | 30 | -87.68 | D-F | 18.11 | (-13.90, 50.12) | 0.266 |
|  | iQOS THP (E) | 30 | -93.98 | A-F | 115.20 | (83.19, 147.21) | <.001 |
|  | Cessation (F) | 30 | -105.79 | C-F | 84.22 | (52.21, 116.23) | <.001 |
|  |  |  |  | E-A | -103.40 | (-135.41, -71.39) | <.001 |
| **Total NNN (ng/24h)** | Non-menthol cigarette (A) | 30 | 0.04 | B-A | -5.75 | (-8.98, -2.51) | <.001 |
|  | Non-menthol glo^TM^ THP (B) | 30 | -5.71 | B-F | 2.25 | (-0.99, 5.48) | 0.172 |
|  | Menthol cigarette (C) | 30 | 1.58 | D-C | -7.58 | (-10.82, -4.35) | <.001 |
|  | Menthol glo^TM^ THP (D) | 30 | -6.00 | D-F | 1.95 | (-1.28, 5.18) | 0.236 |
|  | iQOS THP (E) | 30 | -8.07 | A-F | 7.99 | (4.76, 11.23) | <.001 |
|  | Cessation (F) | 30 | -7.95 | C-F | 9.53 | (6.30, 12.77) | <.001 |
|  |  |  |  | E-A | -8.12 | (-11.35, -4.88) | <.001 |
| **o-tol (ng/24h)** | Non-menthol cigarette (A) | 27 | 23.41 | B-A | -79.15 | (-106.12, -52.17) | <.001 |
|  | Non-menthol glo^TM^ THP (B) | 29 | -55.74 | B-F | 5.89 | (-20.38, 32.15) | 0.659 |
|  | Menthol cigarette (C) | 30 | 4.08 | D-C | -71.58 | (-97.62, -45.54) | <.001 |
|  | Menthol glo^TM^ THP (D) | 30 | -67.50 | D-F | -5.88 | (-31.92, 20.17) | 0.657 |
|  | iQOS THP (E) | 30 | -53.16 | A-F | 85.03 | (58.28, 111.79) | <.001 |
|  | Cessation (F) | 30 | -61.62 | C-F | 65.71 | (39.66, 91.75) | <.001 |
|  |  |  |  | E-A | -76.57 | (-103.33, -49.81) | <.001 |

**Supplementary Material Table 5. Summary of exposure period adverse events.**  Events were coded using MedDRA (Version 19.1). THP, tobacco heating product; AE, adverse event; SAE, serious adverse event; n, number of subjects; ( ), percentage of subjects with adverse events; [ ], number of adverse events; ---, no events reported.

|  | **Product** | | | | | |  |
| --- | --- | --- | --- | --- | --- | --- | --- |
|  | **Non-menthol cigarette**  **(n=30)** | **Non-menthol glo^TM^ THP**  **(n=30)** | **Menthol cigarette**  **(n=30)** | **Menthol glo^TM^ THP**  **(n=30)** | **iQOS THP**  **(n=30)** | **Cessation**  **(n=30)** | **Overall**  **(n=180)** |
| Subjects with AEs | 3 (10.0%) | --- | 1 (3.3%) | 2 (6.7%) | 2 (6.7%) | 2 (6.7%) | 10 (5.6%) |
| Number of AEs | 4 | --- | 2 | 2 | 2 | 4 | 14 |
| Subjects with SAEs | 1 (3.3%)  [1] | --- | --- | --- | --- | --- | 1 (0.6%)  [1] |
| Subjects discontinued due to AEs | --- | --- | --- | --- | --- | --- | --- |
| Severity (all AEs)  Mild | 2 (6.7%)  [3] | --- | 1 (3.3%)  [2] | 1 (3.3%)  [1] | 2 (6.7%)  [2] | 2 (6.7%)  [4] | 8 (4.4%)  [12] |
| Moderate | --- | --- | --- | 1 (3.3%)  [1] | --- | --- | 1 (0.6%)  [1] |
| Severe | 1 (3.3%)  [1] | --- | --- | --- | --- | --- | 1 (0.6%)  [1] |
| Total | 3 (10.0%)  [4] | --- | 1 (3.3%)  [2] | 2 (6.7%)  [2] | 2 (6.7%)  [2] | 2 (6.7%)  [4] | 10 (5.6%)  [14] |
| Severity (possibly related or related to IP)  Mild | --- | --- | --- | --- | --- | --- | --- |
| Moderate | --- | --- | --- | --- | --- | --- | --- |
| Severe | --- | --- | --- | --- | --- | --- | --- |
| Total | --- | --- | --- | --- | --- | --- | --- |

**Supplementary Material Table 6. Frequency of exposure period adverse events for all causalities.** Events were coded using MedDRA (Version 19.1). THP, tobacco heating product; n, number of subjects; ( ), percentage of subjects with adverse events; [ ], number of adverse events; ---, no events reported.

|  | **Product** | | | | | |  |
| --- | --- | --- | --- | --- | --- | --- | --- |
| MedDRA System Organ Class  *Preferred Term* | **Non-menthol cigarette**  **(n=30)** | **Non-menthol glo^TM^ THP**  **(n=30)** | **Menthol cigarette**  **(n=30)** | **Menthol glo^TM^ THP**  **(n=30)** | **iQOS THP**  **(n=30)** | **Cessation**  **(n=30)** | **Overall**  **(n=180)** |
| Overall total | 3 (10.0%)  [4] | --- | 1 (3.3%)  [2] | 2 (6.7%)  [2] | 2 (6.7%)  [2] | 2 (6.7%)  [4] | 10 (5.6%)  [14] |
| Investigations | 2 (6.7%)  [2] | --- | 1 (3.3%)  [2] | 1 (3.3%)  [1] | --- | 2 (6.7%)  [4] | 6 (3.3%)  [9] |
| *Alanine aminotransferase increased* | --- | --- | 1 (3.3%)  [1] | 1 (3.3%)  [1] | --- | 1 (3.3%)  [1] | 3 (1.7%)  [3] |
| *Aspartate aminotransferase increased* | --- | --- | 1 (3.3%)  [1] | --- | --- | 1 (3.3%)  [1] | 2 (1.1%)  [2] |
| *Blood triglycerides increased* | --- | --- | --- | --- | --- | 2 (6.7%)  [2] | 2 (1.1%)  [2] |
| *Glucose urine present* | 1 (3.3%)  [1] | --- | --- | --- | --- | --- | 1 (0.6%)  [1] |
| *Pregnancy test urine positive* | 1 (3.3%)  [1] | --- | --- | --- | --- | --- | 1 (0.6%)  [1] |
| Nervous system disorders | 2 (6.7%)  [2] | --- | --- | 1 (3.3%)  [1] | 2 (6.7%)  [2] | --- | 5 (2.8%)  [5] |
| *Presyncope* | 2 (6.7%)  [2] | --- | --- | 1 (3.3%)  [1] | 2 (6.7%)  [2] | --- | 5 (2.8%)  [5] |
